# Supplementary material for: INI1/SMARCB1 Rpt1 domain mimics TAR RNA in binding to integrase to facilitate HIV-1 replication
Source: Nat Commun. 2021 May 12;12:2743. doi: 10.1038/s41467-021-22733-9 (PMC8115288; doi:10.1038/s41467-021-22733-9)
Supplement: Supplementary file 1 — Supplementary Information [file 41467_2021_22733_MOESM1_ESM.pdf]

Supplementary Table 1, Related to Figure 1:

	<SA>[a]	SA_{lowest}
**Experimental restraints**[b]		
All, Å (1754)	0.122 ± 0.01	0.137
Intraresidue (515)	0.006 ± 0.006	0.075
Sequential (428)	0.197 ± 0.025	0.23
Short (339)	0.035 ± 0.007	0.028
Long (382)	0.136 ± 0.007	0.139
Ambiguous (90)	0.003 ± 0.007	0.001
Hydrogen-bond restraints, Å (58)	0.013 ± 0.003	0.126
Dihedral-angle restraints, ° (116)	0.63 ± 0.39	0.52
NOE violations >0.4 Å	10.7 ± 2.8	11
Angle violations >3°	1.6 ± 1.1	2
**Deviations from idealized covalent geometry**[c]		
Bonds, Å (1348)	0.007 ± 0.001	0.007
Angles, ° (2457)	0.7 ± 0.01	0.68
Improper dihedrals, ° (666)	0.97 ± 0.19	0.89
**Structural statistics for the ensemble**[d]		
PROCHECK parameters		
Most-favored region, %	87.9 ± 2.5	89.3
Additionally allowed, %	9.7 ± 2.2	9.3
Generously allowed, %	0.9 ± 1.4	0.0
Disallowed, %	1.5 ± 1.2	1.3
Number of bad contacts	0.2 ± 0.4	0
**RMSD from the average structure**[E]		
Backbone (N, Ca, C), Å	0.49 ± 0.11	0.41
Heavy atoms, Å	0.93 ± 0.11	0.87

Supplementary Table 1. Statistics for the 20 Lowest Energy NMR Structures of $INI1_{183\text{-}265}$.

[a] <SA> represents the set of 20 selected conformers obtained by restrained dynamical simulated annealing refined in a box of water in CNS/Xplor-NIH. SA_{lowest} refers to the lowest energy structure of the set.

[b]Sum averaging of NOE distance restraints was used for groups with degenerate proton chemical shifts. The interproton unambiguous distance restraint list comprised 515 intraresidue, 428 sequential (|i - j|= 1|), 339 short range (1<|i - j|<5), 382 long-range (|i - j|>5) and 90 ambiguous. Hydrogen-bonds restraints were applied as pairs of distance restraints: HNÂ•Â•Â•O, 1.2-2.2 Å; NÂ•Â•Â•O, 1.2-3.2 Å. The final values for the respective force constants were: NOE, 30 kcal mol-1 Å-2; H-bonds, 50 kcal mol-1 Å-2; dihedral angles, 200 kcal mol-1 rad-2.

[c]The final values for the respective force constants were: bond lengths, 500 kcal mol-1 Å-2; angles and improper torsions, 500 kcal mol-1 rad-2; the improper torsion angle restraints serve to maintain planarity and chirality.

[d]The program PROCHECK (Laskowski et al., 1996) was used to assess the stereochemical parameters of the family of conformers for Ini1. The figures indicate the percentage of residues with backbone φ and ψ angles in separate regions of the Ramachandran plot, defined in the program. The number of bad contacts per 100 residues is expected to be in the range 0-30 for protein crystal structures of >3.0-Å resolution.

[e]The precision of the atomic coordinates is defined as the average pair-wise rmsd between each of the 20 conformers and a mean coordinate structure SA generated by iterative best-fit of the backbone atoms (N, Ca, and C) over residues 183-248 of Ini1 (omitting the flexible C termini residues), followed by coordinate averaging.

Supplementary Table 2, Related to Figure 1:
The table below lists the RMSDs of other NMR and crystal structures as compared to 6AX5 (residues 188-246)

PDB	Cα RMSD (Å)	Backbone RMSD (Å)	Heavyatom RMSD (Å)	Method
5L7B	1.20	1.25	1.98	NMR
5L7A	0.99	1.04	1.78	crystal
5GJK	1.24	1.29	1.83	crystal
6LTJ	1.12	1.15	1.77	CryoEM

Supplementary Table 3, Related to Figure 1:
The table below lists the RMSDs between the Rpt1 structure generated by Robetta and other experimentally resolved structures (residues 188-246)

PDB	Cα RMSD (Å)	Backbone RMSD (Å)	Heavyatom RMSD (Å)	Method
6AX5	1.11	1.16	1.93	NMR
5L7B	0.76	0.79	1.70	NMR
5L7A	0.80	0.85	1.51	crystal
5GJK	0.76	0.77	1.34	crystal
6LTJ	0.80	0.84	1.49	CryoEM

Supplementary Table 4, Related to Figure 7:
The table below illustrates the Comparison of the MDockPP result and the Haddock result (Figure 2) of docking $INI1_{183-304}$ with IN CTD.

	MDockPP	Haddock
ITScorePP score	-359.2	-207.3
Total buried surface area ($Å^2$)	865.0	642.2
Steric clashes	0	0
Electrostatics	good match	good match

Supplementary Table 5, Related to Figure 7:
The table below illustrates the MDockPP results of docking TAR RNA with IN CTD.

	MDockPP
ITScorePP score	-380.5
Total buried surface area ($Å^2$)	826.0
Steric clashes	0
Electrostatics	

Supplementary Table 6
A list of primers and Oligonucleotides:

Supplementary Table 6a: Primers used for cloning INI1$_{183-265}$, INI1$_{183-304}$ and IN fragments	
Primers for amplifying the Vector	VFOR- 5’ - TCCGAATTCGAGCTCCGTCGACAAGC- 3’<br>VREV- 5’ - TCCACCAATCTGTTCTCTGTGAGCCTC- 3’
Primers for amplifying INI1$_{183-265}$ and INI1$_{183-304}$ fragments	S6(Rpt1)-For 5’- GGCTCACAGAGAACAGATTGGTGGATCC ccc gag gtg ctg gtc ccc atc cgg ctg-3’<br>INI1(aa 265)-Rev 5’-GCTTGTCGACGGAGCTCGAATTCGGA**TTA** cta ctt gat gat gac gcg ctg gtc tga--3’<br>INI1(aa 304)-Rev 5’-GCTTGTCGACGGAGCTCGAATTCGGA**TTA** gcc caa ccc cag ctc cga gca cag ctt cag-3’
Supplementary Table 6b: Primers used for mutagenesis of INI1$_{183-304}$ and pCGN-INI1	
Clone	Primer sequence
E3(D225G)	Forward: 5’GCGGGTTCAAATCCAGACCGTCACAGAGGATTTCT-3’<br>Reverse: 5’-AGAAATCCTCTGTGACGGTCTGGATTTGAACCCGC-3’
E4(T214A)	Forward-CATGAATGAGAAGTTGATGGCGCCTGAGATGTTTTCAGA-3’<br>Reverse 5-TCTGAAAACATCTCAGGAGCCATCAACTTCTCATTCATG-3’
E10(D227G)	Forward-5’-CGTCAGCGGGTTCAAACCCAGATCGTCACAGAG-3’<br>Reverse-5’CTCTGTGACGATCTGGGTTTGAACCCGCTGACG-3’
D225E	Forward 5'-GAAATCCTCTGTGACGAGCTGGATTTGAACCCGCT-3'<br>Reverse 5'-AGCGGGTTCAAATCCAGCTCGTCACAGAGGATTTC-3'
Supplementary Table 6c: Primers used for mutagenesis of IN-CTD	
Clone	Primer sequence
IN(W235F)	Forward: 5’-TTTGCTGGTCCTTTGAAAAGTGGATTTCTGCTGTCCCTGTA-3’;<br>Reverse: 5’ TACAGGGACAGCAGAAATCCACTTTTCAAAGGACCAGCAAA-3’
IN-CTD (W235E)	Forward: 5’-GACCTTTGCTGGTCCTTTCTCAAGTGGATTTCTGCTGCTC-3’;<br>Reverse: 5’GGACAGCAGAAATCCACTTGAGAAAGGACCAGCAAAGCTC-3’
IN-CTD (W235A)	Forward: 5’-GAGCTTTGCTGGTCCTTTCGCAAGTGGATTTCTGCTGCTC-3’;<br>Reverse: 5’-GGACAGCAGAAATCCACTTGCGAAAGGACCAGCAAAGCTC-3’
IN-CTD (W235K)	Forward: 5’-GGACAGCAGAAATCCACTTAAGAAAGGACCAGCAAAGCTC-3’;<br>Reverse: 5’-GAGCTTTGCTGGTCCTTTCTTAAGTGGATTTCTGCTGCTC-3’
IN-CTD (W235F)	
IN-CTD (K264A/ K266A)	Forward- 5’ CCATCTGTTTTCCATAATCCCTAATGATCGCTGCTGCTCTTCTTGGCACTACTTTTATGTCACTAT-3’; Reverse-5’ATAGTGACATAAAAGTAGTGCCAAGAAGAGCAGCAGCGATCATTAGGGATTATGGAAAACAGATGG-3’
IN-CTD (K269A/ K273A)	Forward- 5’-CACCTGCCATCTGTGCTCCATAATCCGCAATGATCTTTGCTTTTCTTCTTGGCAC-3’<br>Reverse-5’-GTGCCAAGAAGAAAAGCAAAGATCATTGCGGATTATGGAGCACAGATGGCAGGTG-3’
IN-CTD (R228A)	Forward: - 5’ CAA AGT GGA TTT CTG CTG TCC GCG TAA TAA ACC CGA AAA TTT TGA ATT TTT G - 3’<br>Reverse: -CAA AAA TTC AAA ATT TTC GGG TTT ATT ACG CGG ACA GCA GAA ATC CAC TTT G - 3’
IN-CTD (K244A)	Forward: - CTA CTG CCC CTT CAC CTG CCC AGA GGA GCT TTG CTG<br>Reverse: - CAG CAA AGC TCC TCT GGG CAG GTG AAG GGG CAG TAG
Supplementary Table 6d: Sequence of the TAR RNA oligonucleotide	
RNAs	Sequence
Biotin labelled TAR (HIV-1 vRNA(1-57)-BIO-TAR)	5’ -biotin dT/GGUCUCUCUGGU UAGACCAGAUCUGAGCCUGGGAGCUCUCUGGCUA ACUAGGGAACC/3’ -biotin dT
Unlabeled TAR (HIV-1 vRNA(1-57)-TAR):	5’ GGUCUCUCUGGUUAGACCAGA UCUGAGCCUGGGAGCUCUCUGGCUAACUAGGG AACC 3’
Unlabeled Control RNA (HIV-1 vRNA(237-279):	5’ GCAGGACUCGGCUUGCUGAA GCGCGCACGGCAAGAGGCGAGGG 3’
Supplementary Table 6e: primers and probes used to detect various stages of HIV-1 replication	
Early RT primers	ert2f 5’-GTGCCCGTCTGTTGTGTGAC-3’,<br>ert2r 5’-GGCGCCACTGCTAGAGATTT-3’, and<br>ert2 probe 5’-CTAGAGATCCCTCAGACCCTTTTAGTCAGTGTGG-3’.
Late RT primers,	MH531 forward 5’-TGTGTGCCCGTCTGTTGTGT-3’,<br>MH532 reverse 5’-GAGTCCTGCGTCGAGAGAGC-3’, and<br>LRT probe 5’-CAGTGGCGCCCGAACAGGGA-3’.
2LTR circles	MH535 forward 5’-AACTAGGGAACCCACTGCTTAAG-3’,<br>MH536 reverse 5’-TCCACAGATCAAGGATATCTTGTC-3’, and<br>MH603 probe 5’-ACACTACTTGAAGCACTCAAGGCAAGCTTT-3’.
Alu-gag primers	Alu forward 5′- GCC TCC CAA AGT GCT GGG ATT ACA G -3′<br>HIV gag (Reverse) nucleotides (nt) 1505-1486 5′ - GTT CCT GCT ATG TCA CTT CC – 3′

Supplementary Fig. 1: Related to Figure 1

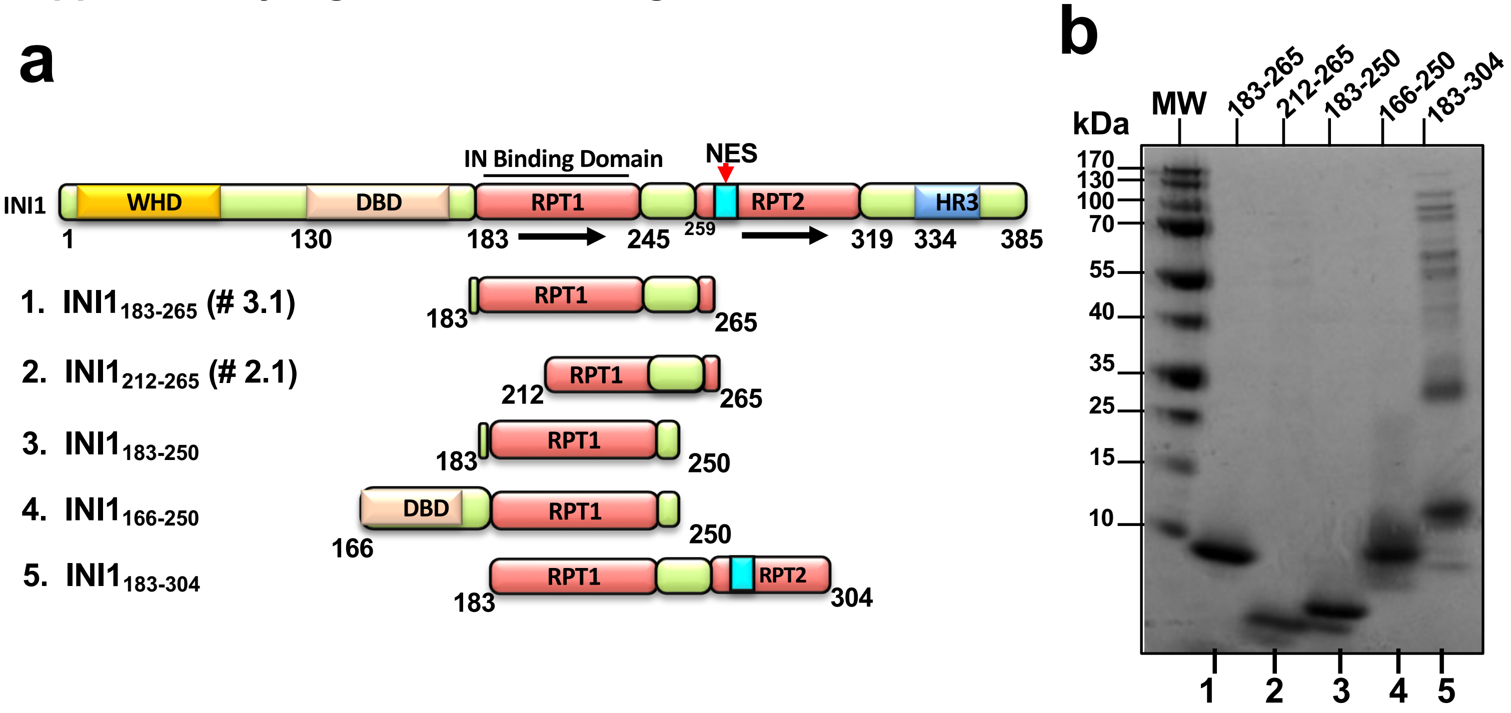

Supplementary Fig. 1: Cloning, expression and purification of overlapping fragments of INI1 containing Rpt1. **a** Cartoon representing various overlapping fragments of INI1. Numbers below the bars represent amino acid residue positions. The top bar represents full length INI1, and the bars 1-5 below the top bar represents INI1 fragments. Number in the parentheses indicate clone numbers. WHD (in yellow) = Winged Helix DNA binding domain; DBD (in cream) = DNA Binding Domain; RPT (in red) = Repeat; NES (in turquoise) = Nuclear Export Signal; HR3 (in blue) = homology region 3; and arrows represent repeats. **b** Coomassie stained SDS/PAGE gel indicating purified INI1 fragments from one out of three independent experiments. The fragments were cloned as His_6-SUMO-fusions and were purified in two steps using Ni-NTA column as described in the methods for purification of $INI1_{183-265}$. Lane numbers correspond to the fragments in ***a***.

Supplementary Fig. 2: Related to Figure 1

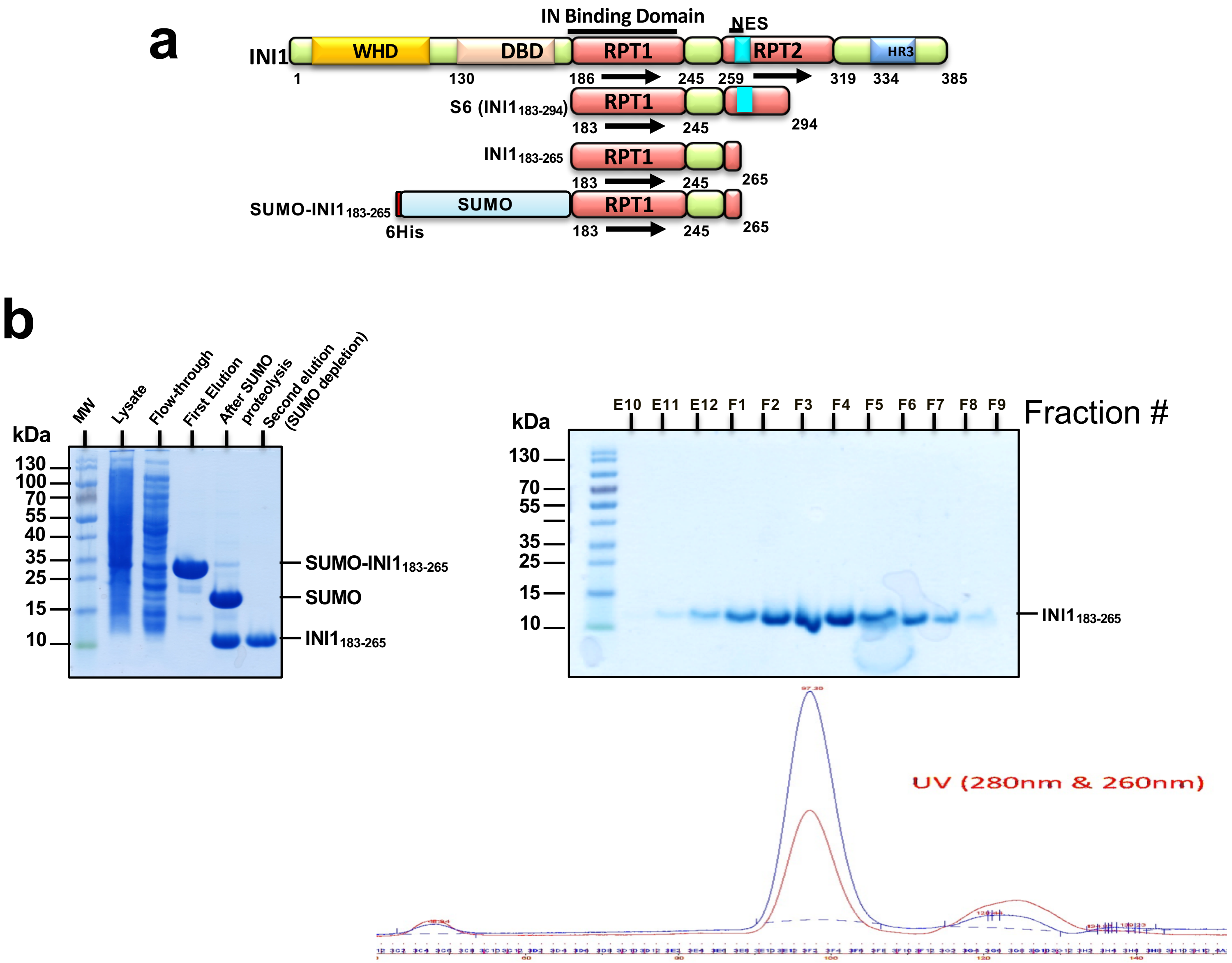

Supplementary Fig. 2: **Purification of INI1$_{183\text{-}265}$ fragment.** **a** Schematic representation of INI1 (top bar) and INI1 fragments, the transdominant negative mutant S6(INI1$_{183\text{-}294}$), INI1$_{183\text{-}265}$ fragment, and the Sumo fusion of INI1$_{183\text{-}265}$ fragment. Numbers below the bar represent amino acid positions. WHD (in yellow) = Winged Helix DNA binding domain; DBD (in cream) = DNA Binding Domain; RPT (in red) = Repeat; NES (in turquoise) = Nuclear Export Signal; HR3 (in blue) = homology region 3; and arrows represent repeats. **b** Coomassie gel indicating proteins from various stages of purification of INI1$_{183\text{-}265}$ as indicated in the labels for the lanes (representative gels from one out of three independent experiments is shown). The left panel indicates two-step purification of INI1$_{183\text{-}265}$. The right panel indicates peak fractions collected from subsequent gel filtration chromatography. The numbers above the lanes indicate fraction numbers. The graph below the right panel indicates the absorbance (at 280 and 260 nm) of the fractions collected from the gel filtration column.

Supplementary Fig. 3: Related to Figure 1

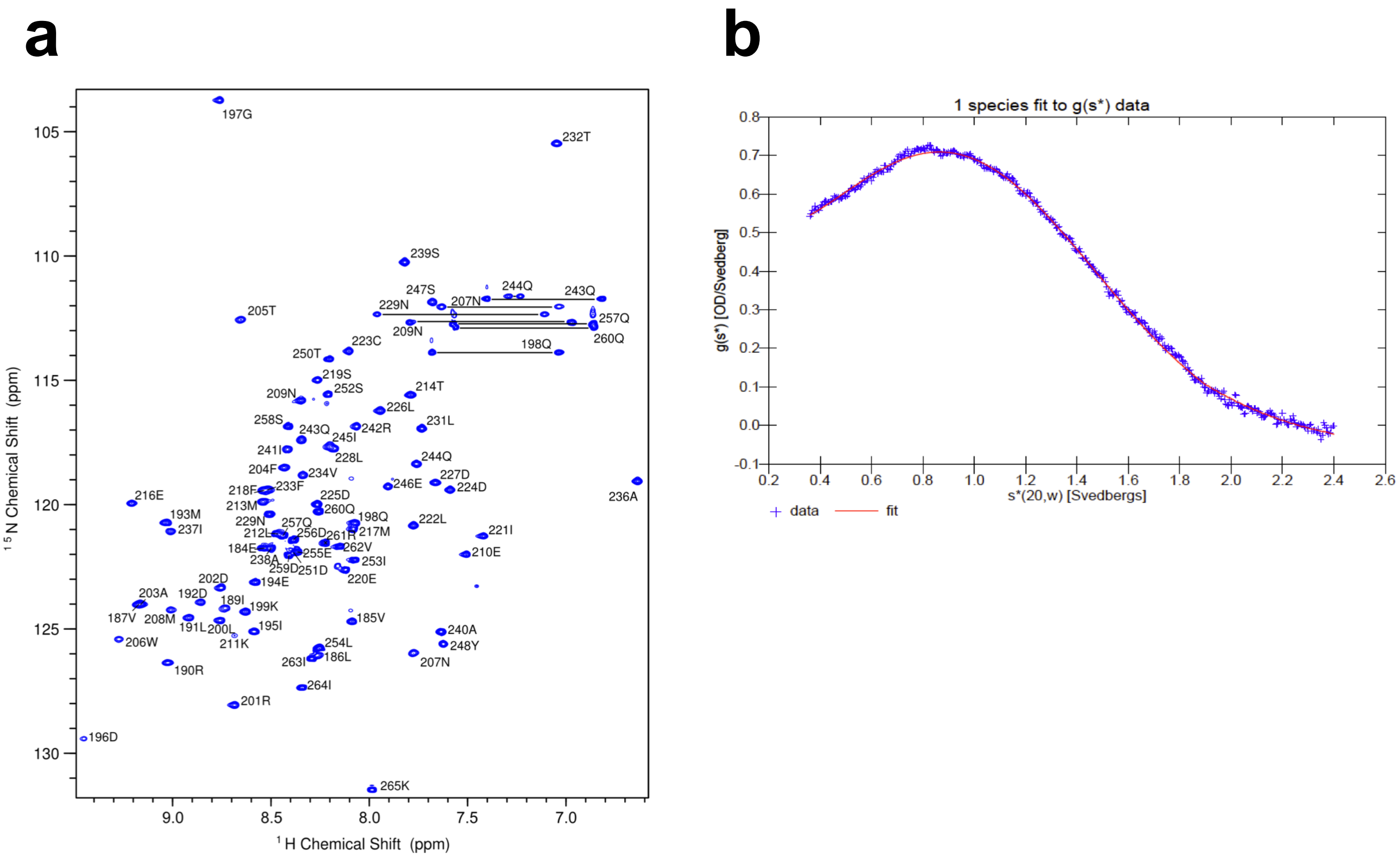

Supplementary Fig. 3: ^{1}H^{15}N HSQC NMR spectrum of 1mM INI1$_{183\text{-}265}$ and analytical Ultra Centrifugation of INI1$_{183\text{-}265}$. a Time-derived distribution of INI1$_{183\text{-}304}$ determined as described below. **b** Analytical ultra centrifugation. The crosses denotes the data points. The line denotes the best fit to the noninteracting single component model. Forty four of the 120 sedimentation scans were used to calculate the time-derivative distribution. INI1$_{183\text{-}265}$ sediments as a single component characterized by $S_{20,w}$ = 1.035 (1.033, 1.038) *S* and an apparent molecular weight $(S/D)_{20,w}$ = 9.92 (9.55, 10.09) *kDa*. The diffusion coefficient corresponding to the best fit molecular mass is 9.66 *F*. Since the molecular weight calculated from the sequence of INI1$_{183\text{-}265}$ is 9.566 *kDa*, we conclude that under the solution conditions analyzed this protein is monomeric.

Supplementary Fig. 4: Related to Figure 1

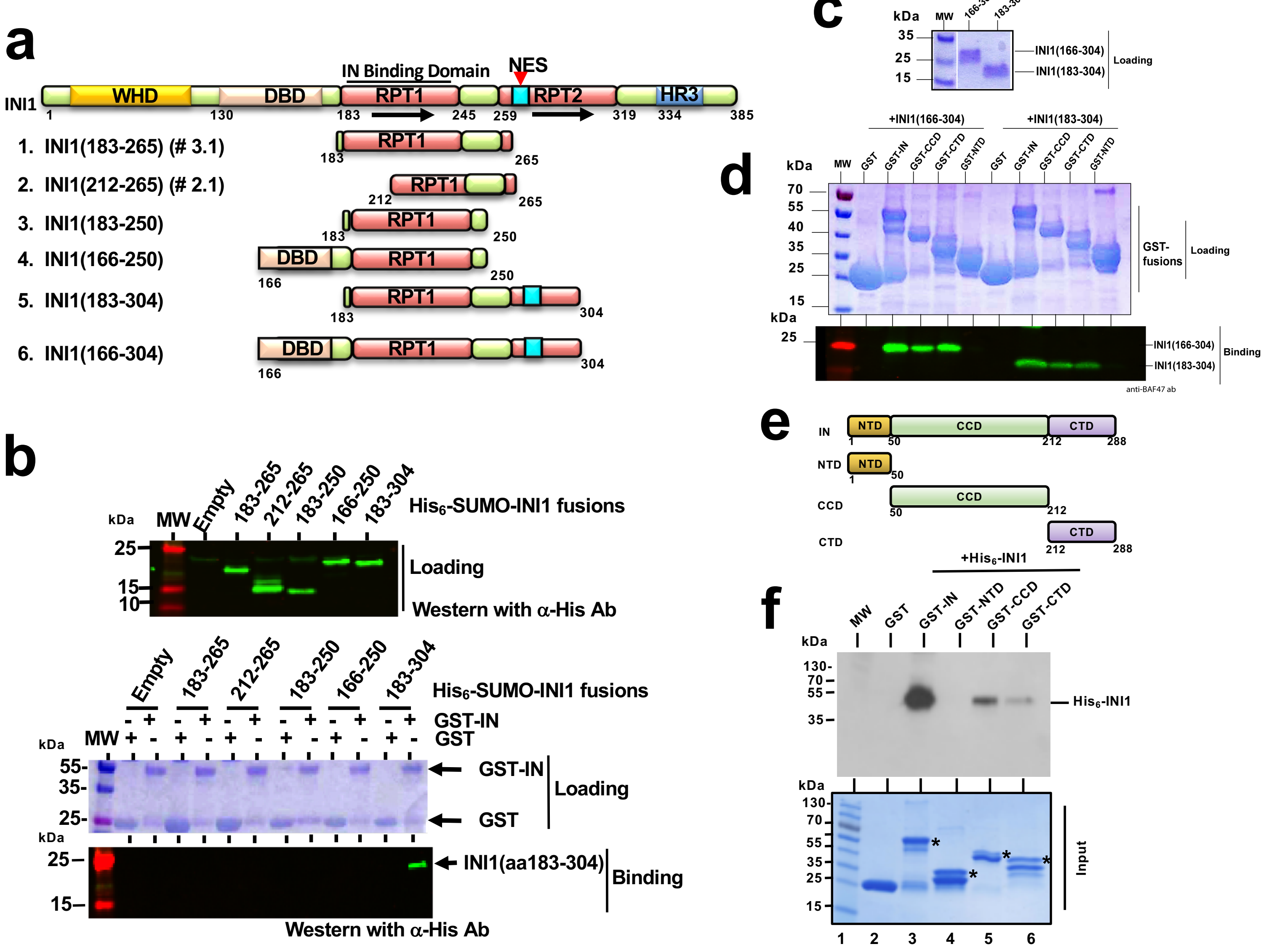

Supplementary Fig. 4: *In vitro* binding of IN and its domains with INI1 and its fragments. a Cartoon representing various INI1 fragments (1-6) as in Supplementary Fig 1a, all of which were expressed as His$_6$-SUMO fusions and used for *in vitro* binding with IN as in ***b-c***. **b** Results of *in vitro* binding of various His$_6$-SUMO-INI1 fragments with GST-IN. *Top panel*: Western blot of lysates expressing INI1 fragments used for binding experiment; *Middle panel*: Loading control for GST-IN and GST proteins; *Bottom panel*: Bound INI1 fragments. Note the positive interaction of INI1$_{183-304}$ fragment in the last lane. **c-d** Binding of purified INI1$_{183-304}$ and INI1$_{166-304}$ fragments with GST-fusion of IN and domains: GST-pull down assay was carried out as described in the methods and bound proteins were detected using α-BAF47 antibodies. **c** Coomassie blue stained gel of INI1$_{183-304}$ and INI1$_{166-304}$ proteins loading control. **d** Top panel, Coomassie blue stained gel of GST fusions of IN, central core, C-terminal and N-terminal domains (CCD, CTD and NTD) as loading control. Bottom panel, Immunoblot of the bound proteins, INI1$_{183-304}$ and INI1$_{166-304}$ fragments. **e-f** *In vitro* binding of domain of IN with full-length INI1. **e** Cartoon representing IN and its three domains. **f** Top panel, Immunoblot of the bound INI1. Bottom panel, GST-fusions of IN and domains with His$_6$-INI1 as input control. The "*" indicates the position of full length protein in each case. In the panels ***b-d*** and ***f*** representative images from one out three independent experiments are shown. "MW" is molecular weight markers.

Supplementary Fig. 5: Related to Figure 1

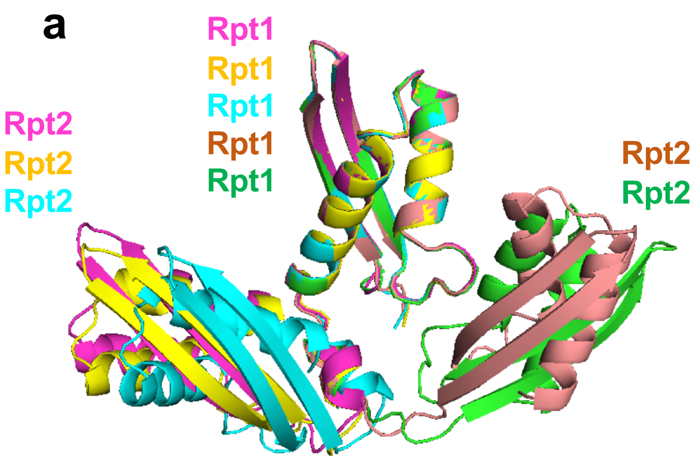

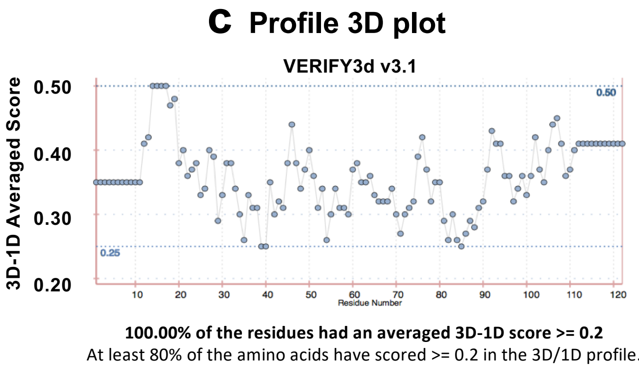

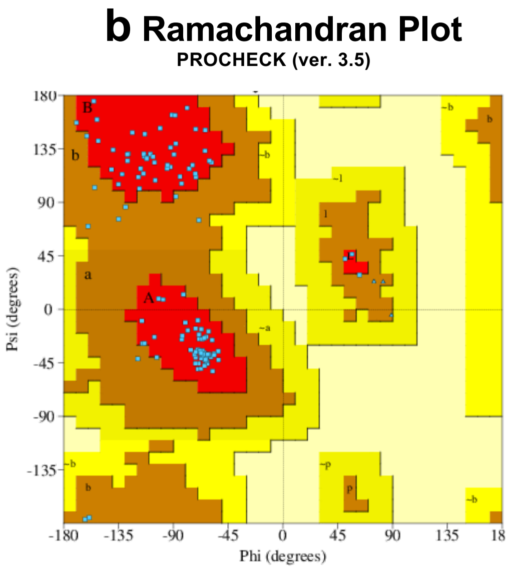

	No. of residues	%
Most favoured regions	100	90.1%
Additional allowed regions	11	9.9%
Generously allowed regions	0	0.0%
Disallowed regions	0	0.0%
Non-glycine and non-proline residues	111	100.0%
Glycine residues	4	
Proline residues	7	
Total number of residues	122	

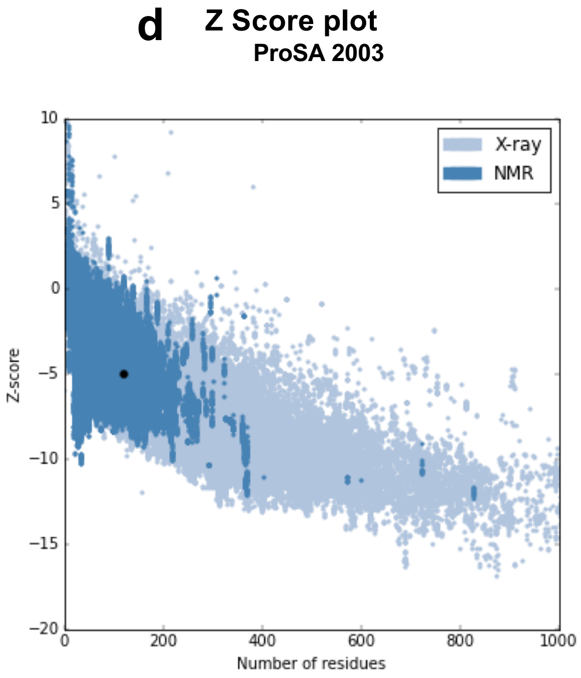

Supplementary Fig. 5: Modeling of INI1$_{183\text{-}319}$ fragment containing Rpt1-linker-Rpt2 regions and validation of INI1$_{183\text{-}304}$ model. a Ribbon diagram representing the superimposition of representative INI1$_{183\text{-}319}$ models from five different clusters obtained from Robetta. These five models are color coded in pink, yellow, turquoise, brown and green colors. Note that while Rpt1 region of each model is perfectly aligned, the spatial positioning of the Rpt2 in relation to Rpt1 is altered due to the differential folding of the linker region. While two Rpt2 models (brown and green) occupy space right of the Rpt1 the other three Rpt2 models (pink, yellow and turquoise), the left side of Rpt1. **b-d** Validation of the modeling of INI1$_{183\text{-}304}$ structure. **b** The Ramachandran plot indicating the presence of 90.1% and 9.9% residues in the favored and allowed regions, and 0% residues in the outlier region, respectively. **c** Profile 3D plot (using VERIFY3d v3.1) to determine how well the structure is folded in the 3D space. **d** Z score plot (obtained using ProSA 2003) to determine the energetics of the modelled protein.

Supplementary Fig. 6: Related to Figures 2 and 3

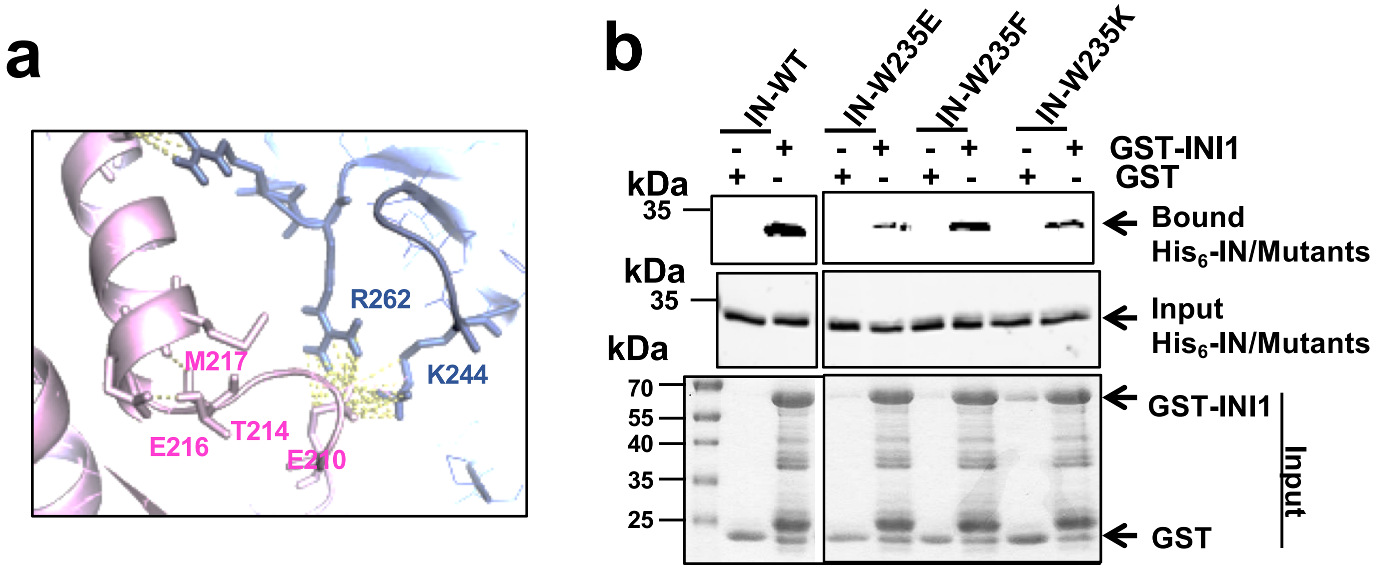

Supplementary Fig. 6: a An exploded view of T214 residue in the IN-CTD/INI1$_{183\text{-}304}$ complex structure; and *In vitro* binding of His$_6$-fusions of IN and mutants W235E, W235F and W235K with GST-INI1. a INI1-Rpt1 portion and its residues are indicated in pink and IN-CTD portion and its residues are indicated in blue. **b** The *in vitro* binding assay was carried out by using the bacterial lysates expressing His$_6$-IN or its mutants and GST-INI1 immobilized on G-beads. The top panel represents the Western blot of the bound proteins, middle panel represents Western blot of the input control of His$_6$-IN fusion proteins and the bottom panel represents the input control of the GST-INI1 fusion proteins. (Please note: Uncropped gels are provided in the Supplementary data files).

Supplementary Figure 7: Related to Figure 4

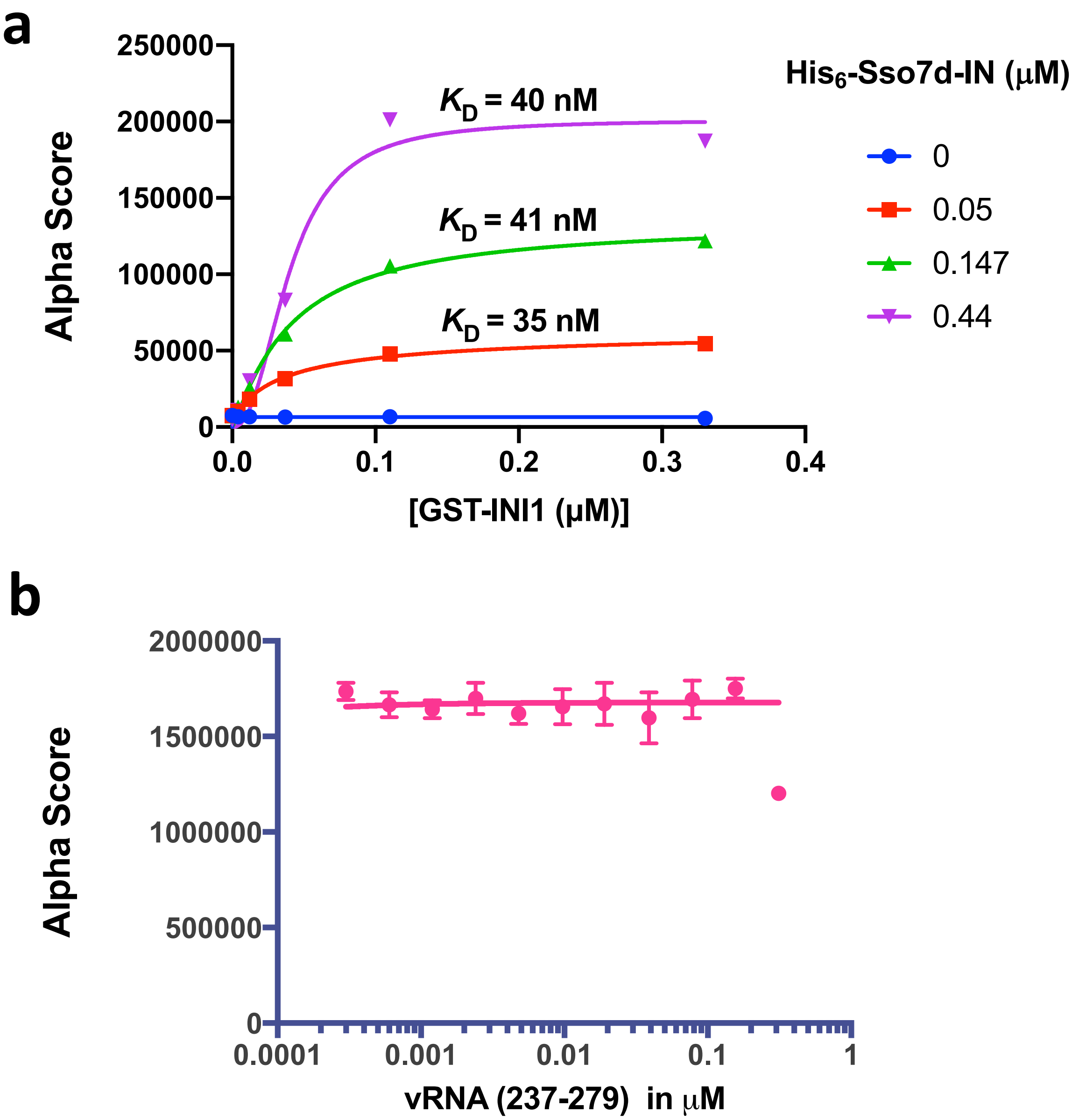

Supplementary Figure 7: a Alpha assay to determine the interaction between full length IN/INI1 and Competition of IN-CTD and INI1$_{183\text{-}304}$ interaction with viral RNA nucleotides, **ntd (237-279): a** Interaction of His$_6$-Sso7d-IN with GST-INI1. The graph represents one representative experiment. Increasing concentrations of GST-INI1 were incubated with fixed concentration of His$_6$-Sso7d-IN and the interactions were detected as Alpha Score. The K_D values were determined by nonlinear regression analysis using specific binding with Hill slope analysis in GraphPad Prism. **b** Increasing concentration of HIV-1 **v**iral RNA fragment from the region 237-279 was used to test its effect on binding of IN-CTD with INI1$_{183\text{-}304}$. Note that the IN-CTD/INI1$_{183\text{-}304}$ interactions were not significantly inhibited by HIV-1 RNA ntd (237-279). The graph is derived from plotting values from six independent experiments (*n*=6 independent experiments). The graph represents Mean+/-SEM.

Supplementary Figure 8: Related to Figure 6

a

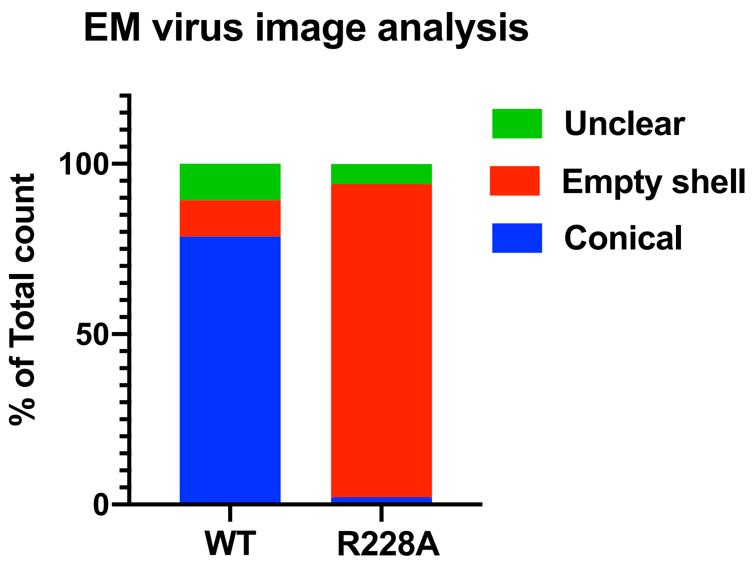

b

	Conical core		Non-conical core		Dual or nested cores		
	No eccentric condensate	Eccentric condensate	No eccentric condensate	Eccentric condensate	No eccentric condensate	Eccentric condensate	Immature
WT (n=23)	15 (65.2%)	1 (4.3%)	0 (0%)	1 (4.3%)	1 (4.3%)	4 (17.4%)	1 (4.3%)
IN W235E (n=130)	30 (23.1%)	30 (23.1%)	2 (1.5%)	20 (15.4%)	9 (6.9%)	38 (29.2%)	1 (0.8%)

Supplementary Figure 8: Quantitation of virus particle morphology of wild type and mutant IN virions. a Graphical representation of % of normal (conical) and abnormal particles in the Transmission Electron Microscopy analysis of WT and W235E mutant. While the ”conical” in the legend represents the wild type virion particles, “empty shell” represents the lack of electron dense material within the capsid core and presence of eccentric accumulation of electron dense materials. (WT, n= 103 particles; and R228A n= 186 particles**). b** Table depicts the % of various normal and defective particles identified in WT and W235E mutant using the CryoET analysis.

Supplementary Figure 9: Related to Figure 7

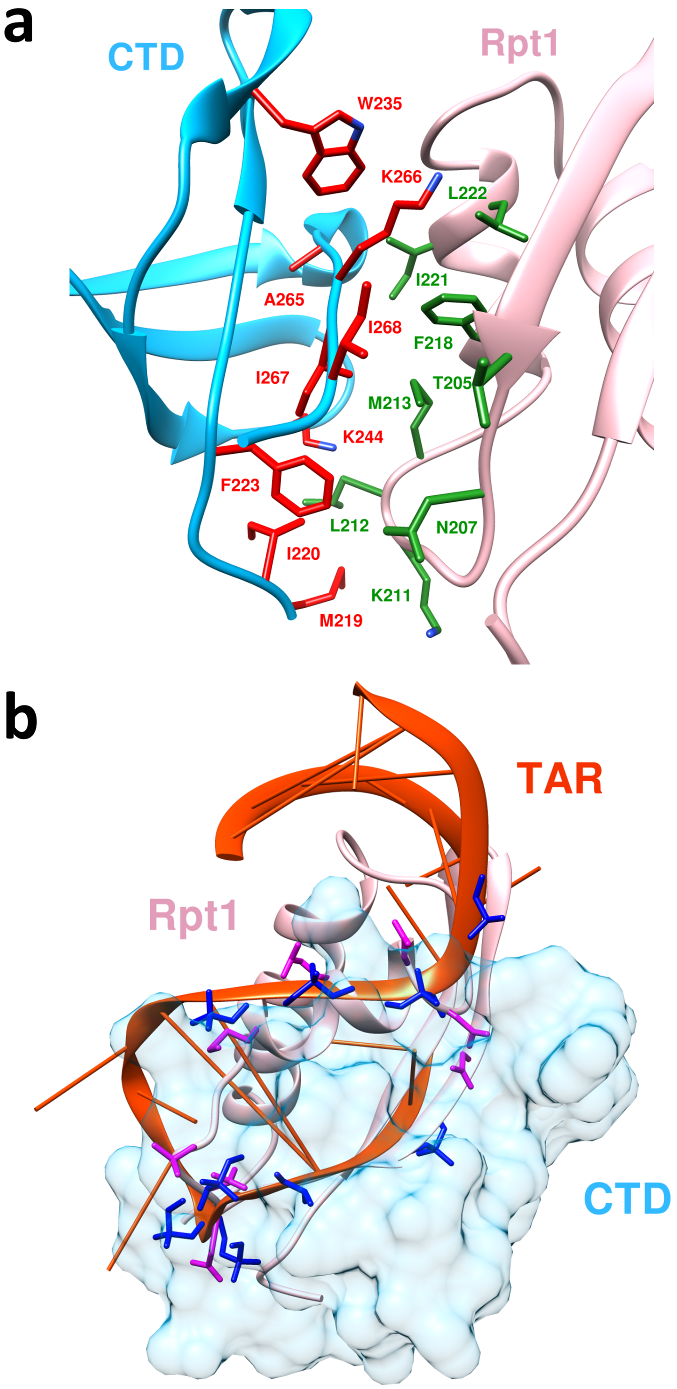

Supplementary Figure 9: Modeling of IN-CTD/INI1$_{183\text{-}304}$ and IN-CTD/TAR RNA interactions using MDockPP. a Ribbon diagram of the exploded view of IN-CTD/INI1$_{183\text{-}304}$ complex structure model generated by MDockPP to show the presence of hydrophobic patch in the interface residues. INI1 portion is indicated in pink and INI1 residues in green; and CTD portion is indicated in blue and CTD residues in red. **b** Superimposition of docking complex models of IN-CTD/INI1$_{183\text{-}304}$ and IN-CTD/TAR generated by MDockPP. INI1 portion is indicated in pink, RNA in Orange and three-dimensional surface of CTD portion in light blue. Please note the close proximity of phosphate groups of TAR (in Dark Blue) nucleotides with negatively charged residues of INI1$_{183\text{-}304}$ (in Magenta), both of which are involved in interacting with CTD.
