# Supplementary material for: INI1/SMARCB1 Rpt1 domain mimics TAR RNA in binding to integrase to facilitate HIV-1 replication
Source: Nat Commun. 2021 May 12;12:2743. doi: 10.1038/s41467-021-22733-9 (PMC8115288; doi:10.1038/s41467-021-22733-9)
Supplement: Supplementary file 3 — Description of Additional Supplementary Files [file 41467_2021_22733_MOESM3_ESM.pdf]

Description of Additional Supplementary Files

File Name: Supplementary Data 1
Description: The content of the "Supplementary Data 1" include nine .pdb files of models generated in this report. The corresponding figure numbers are included in the file names.

- Fig1e_INI1(183-304)robetta-1.pdb
- Fig2_INI1(183-304)_CTD_docked_no restrinsts_Savita.pdb
- Fig7ab_INI1(183-304)_CTD_docked_with restraints-Liming.pdb
- Fig7cd_TARRAN_CTD_docked_with restraints-Liming.pdb
- SuppleFig5a_Fullrpt1rpt2model1.pdb
- SuppleFig5a_Fullrpt1rpt2model2.pdb
- SuppleFig5a_Fullrpt1rpt2model3.pdb
- SuppleFig5a_Fullrpt1rpt2model4.pdb
- SuppleFig5a_Fullrpt1rpt2model5.pdb
