# Supplementary material for: INI1/SMARCB1 Rpt1 domain mimics TAR RNA in binding to integrase to facilitate HIV-1 replication
Source: Nat Commun. 2021 May 12;12:2743. doi: 10.1038/s41467-021-22733-9 (PMC8115288; doi:10.1038/s41467-021-22733-9)
Supplement: Supplementary file 5 — Reporting Summary [file 41467_2021_22733_MOESM5_ESM.pdf]

nature research

Corresponding author(s): Ganjam V. Kalpana

Last updated by author(s): 03/12/2021

Reporting Summary

Nature Research wishes to improve the reproducibility of the work that we publish. This form provides structure for consistency and transparency in reporting. For further information on Nature Research policies, see our Editorial Policies and the Editorial Policy Checklist.

Statistics

For all statistical analyses, confirm that the following items are present in the figure legend, table legend, main text, or Methods section.

n/a	Confirmed	
☐	☒	The exact sample size (*n*) for each experimental group/condition, given as a discrete number and unit of measurement
☐	☒	A statement on whether measurements were taken from distinct samples or whether the same sample was measured repeatedly
☒	☐	The statistical test(s) used AND whether they are one- or two-sided<br>*Only common tests should be described solely by name; describe more complex techniques in the Methods section.*
☒	☐	A description of all covariates tested
☒	☐	A description of any assumptions or corrections, such as tests of normality and adjustment for multiple comparisons
☐	☒	A full description of the statistical parameters including central tendency (e.g. means) or other basic estimates (e.g. regression coefficient) AND variation (e.g. standard deviation) or associated estimates of uncertainty (e.g. confidence intervals)
☒	☐	For null hypothesis testing, the test statistic (e.g. *F*, *t*, *r*) with confidence intervals, effect sizes, degrees of freedom and *P* value noted<br>*Give P values as exact values whenever suitable.*
☒	☐	For Bayesian analysis, information on the choice of priors and Markov chain Monte Carlo settings
☒	☐	For hierarchical and complex designs, identification of the appropriate level for tests and full reporting of outcomes
☒	☐	Estimates of effect sizes (e.g. Cohen's *d*, Pearson's *r*), indicating how they were calculated

Our web collection on statistics for biologists contains articles on many of the points above.

Software and code

Policy information about availability of computer code

Data collection	1) NMR: NMR data were collected with the manufacturer's TopSpin v2.3 operating software.<br>2) Protein and RNA modeling: Protein structure modeling was performed using Robetta, a protein structure prediction server accessible at https://robetta.bakerlab.org, which is curated by David Baker's laboratory. The protein-protein and protein-RNA structure predictions were performed using two docking servers: One is HADDOCK, which is developed and maintained by Bovin's laboratory at https://wenmr.science.uu.nl/haddock2.4. The other is our in-house docking server, MDockPP, for protein-protein and protein-RNA structure prediction [Xu, X. et al. Performance of MDockPP in CAPRI rounds 28-29 and 31-35 including the prediction of water-mediated interactions. Proteins 85, 424-434 (2017)], and is accessible at http://zougrouptoolkit.missouri.edu/MDockPP.<br>3) CryoEM: Tilt series were acquired using SerialEM v3.5 [Mastronarde DN. Automated electron microscope tomography using robust prediction of specimen movements. J Struct Biol. 2005 Oct;152(1):36-51. doi: 10.1016/j.jsb.2005.07.007. PMID: 16182563].
Data analysis	1) NMR: Non-uniform sampled data was processed using the MDDNMR v2.1 package [Kazimierczuk, K. & Orekhov, V.Y. Accelerated NMR spectroscopy by using compressed sensing. Angew Chem Int Ed Engl 50, 5556-9 (2011)]. General transformation from time domain data used NMRPIPE [Delaglio, F. et al. NMRPipe: a multidimensional spectral processing system based on UNIX pipes. J Biomol NMR 6, 277-93 (1995)]. Assignment and structure determination followed the standard procedures in CCPN Analysis 2,3 [Skinner, S.P. et al. CcpNmr AnalysisAssign: a flexible platform for integrated NMR analysis. J Biomol NMR 66, 111-124 (2016)]. NMR structure quality was assessed using the standard PDB processes (https://www.rcsb.org/#Subcategory-analyze_quality).<br>2) Protein and RNA modeling: Software tools used in data analysis include both publicly available software and in-house programs. Specifically, the quality of the modeled protein structure through Robetta was evaluated with PROCHECK v3.5 [Laskowski, R.A., Rullmannn, J.A., MacArthur, M.W., Kaptein, R. & Thornton, J.M. AQUA and PROCHECK-NMR: programs for checking the quality of protein structures solved by NMR. J Biomol NMR 8, 477-86 (1996)], WHAT IF v8.0 [Vriend, G. WHAT IF: a molecular modeling and drug design program. J Mol Graph 8, 52-6, 29 (1990)], VERIFY3D v3.1 [Eisenberg, D., Luthy, R. & Bowie, J.U. VERIFY3D: assessment of protein models with three-dimensional profiles. Methods Enzymol 277, 396-404 (1997)], and PROSA 2003 [Wiederstein, M. & Sippl, M.J. ProSA-web: interactive web service for the recognition of errors in three-dimensional structures of proteins. Nucleic Acids Res 35, W407-10 (2007)]. The generated protein-protein and

protein-RNA complex structures were ranked by the scoring functions in HADDOCK (https://wenmr.science.uu.nl/haddock2.4), ITScanPR [Huang, S.Y. & Zou, X. A knowledge-based scoring function for protein-RNA interactions derived from a statistical mechanics-based iterative method. Nucleic Acids Res 42, e55 (2014)] and in-house MDockPP (http://zougrouptoolkit.missouri.edu/MDockPP).
3) Structure visualization, structure characterization and analysis, and image rendering: CCP4mg (https://www.ccp4.ac.uk/MG/), MacPymol (PyMOL v1.7.6.4 Enhanced for Mac OS X, https://pymol.org/), Maestro (Schrodinger, https://www.schrodinger.com/maestro), and UCSF Chimera (version 1.14 at https://www.cgl.ucsf.edu/chimera).
3) CryoEM: Cryo-electron tomograms were reconstructed using Bsoft v1.8 [Heymann JB, Cardone G, Winkler DC, Steven AC. Computational resources for cryo-electron tomography in Bsoft. J Struct Biol. 2008;161(3):232-242. doi:10.1016/j.jsb.2007.08.002].
4) Results were analyzed using Graph Pad Prism 9 version 9.0.0. (GraphPad software, CA).

For manuscripts utilizing custom algorithms or software that are central to the research but not yet described in published literature, software must be made available to editors and reviewers. We strongly encourage code deposition in a community repository (e.g. GitHub). See the Nature Research guidelines for submitting code & software for further information.

Data

Policy information about availability of data

All manuscripts must include a data availability statement. This statement should provide the following information, where applicable:

- Accession codes, unique identifiers, or web links for publicly available datasets
- A list of figures that have associated raw data
- A description of any restrictions on data availability

A reporting summary for this article is available as Supplementary Information file. The main data supporting the findings of this study are available within the article and its Supplementary Figures. The structures in the Figs.1b,c and Figs.8a,b are derived from the NMR structure deposited in the PDB database with ID:6AX5 (https://www.rcsb.org/structure/6AX5). The structural data for PDB IDs: 5I7A, 5I7B, 5GJK, 6LTJ, 1QMC and 1ANR used in the Fig.1d are available in the PDB database (https://www.rcsb.org/). The PDB files of the modeling and docking data underlying Fig.1e, Fig2, and Fig.7 are provided as Supplementary data. The source data underlying Figs. 3-5, and Fig. 6c-j are provided as a Source Data file. The data in the Fig. 6b are deposited in the EMBD database (under entry IDs EMD-22410 and EMD-22411). MDockPP structure prediction software used for prediction of strutures of docked complexes in the Fig.7 is accessible at http://zougrouptoolkit.missouri.edu/MDockPP. Source data and Supplementary data are provided with this paper.

Field-specific reporting

Please select the one below that is the best fit for your research. If you are not sure, read the appropriate sections before making your selection.

- [x] Life sciences
- [ ] Behavioural & social sciences
- [ ] Ecological, evolutionary & environmental sciences

For a reference copy of the document with all sections, see nature.com/documents/nr-reporting-summary-flat.pdf

Life sciences study design

All studies must disclose on these points even when the disclosure is negative.

Sample size	The biochemical and cell biology experiments were independently conducted three times to assess experimental variation and to more precisely estimate the mean. Three replicates are considered sufficient to increase precision given each experiment was conducted in an identical manner from three different preparations of the same protein (WT or mutants), and technical variability was assumed to be very low. No formal statistical comparison across proteins was performed.
Data exclusions	No data were excluded.
Replication	All experiments were conducted at least three independent times using independently prepared biological replicates to ensure reproducibility. The mean of the data was plotted with standard error. All the attempts at replication were successful.
Randomization	This is not relevant to our study as it does not include the analysis of large sample size and each test sample had just one variable. For example, wild type protein was compared to a mutant protein derived from the same wild type protein and prepared in an identical manner. A negative and a positive controls were used to compare to test samples. As we are not allocating samples into different experimental groups, randomization is not applicable here.
Blinding	Blinding was used whenever possible such as when analyzing the Electron microscopy data. However complete blinding is not possible with these experiments. There was no allocation of samples to groups. All experiments were performed and assessed in an identical manner; the potential for bias is minimal.

Reporting for specific materials, systems and methods

We require information from authors about some types of materials, experimental systems and methods used in many studies. Here, indicate whether each material, system or method listed is relevant to your study. If you are not sure if a list item applies to your research, read the appropriate section before selecting a response.

Materials & experimental systems

n/a	Involved in the study
☐	☒ Antibodies
☐	☒ Eukaryotic cell lines
☒	☐ Palaeontology and archaeology
☒	☐ Animals and other organisms
☒	☐ Human research participants
☒	☐ Clinical data
☒	☐ Dual use research of concern

Methods

n/a	Involved in the study
☒	☐ ChIP-seq
☒	☐ Flow cytometry
☒	☐ MRI-based neuroimaging

Antibodies

Antibodies used

1) Mouse anti-INI1(BAF47) Ab: BD Transduction laboratories (Catalogue # 612110; Lot # 7144795; 1:1000 dilution).
2) Mouse anti-HA Ab: Santa Cruz (Catalogue # SC-7392; Lot # L1218; 1:1000 dilution).
3) Rabbit anti-GFP Ab: Cell Signal (Catalogue # 2555; Lot # 8; 1:1000 dilution).
4) Goat anti-p24 Ab: A gift of Dr. David Ott, NIH (Lot #000938; 1:1000 dilution).
5) Rabbit anti-IN Ab: NIH AIDS reagent and repository (Catalogue # 3514; Lot # 130353, 1:500 dilution).
6) anti-6His antibody : Clontech (Catalogue # 631212; Lot# 8071803; 1:1000 dilution)
7) Rabbit polyclonal isotype IgG antibody: Santa Cruise Biotechnology (Catalogue # SC-51993; Lot # F0316; 1:1000 dilution).

Validation

These antibodies have been validated at the manufacturing source by routinely testing the antibodies for detection of a correct size band on a Western from a cell line and/or from purified proteins. These antibodies were also validated in our laboratory by testing their ability to detect the correct size band in a Western of a purified protein and/or cell lysate. Negative and positive controls were used while testing these antibodies. For testing anti-INI1 antibodies, 293T (INI1+/+) cell lines was used as positive control and MON (INI1-/-) cell line was included as the negative control. For testing the remaining antibodies, 293T cell lysates were used as negative control and 293T cells expressing either YFP-IN or HA-INI1 were used as positive controls for testing anti-GFP or anti-HA antibodies. To test the anti-IN antibodies lysates from uninfected 293T cells were used as negative control and HIV-1 infected cells were used as positive control. Isotype IgG antibody was validated by its inability to immunoprecipitate GFP, GFP-IN or HA-INI1 proteins from the cell lysates.

Eukaryotic cell lines

Policy information about cell lines

Cell line source(s)

1) 293T/17 SF [HEK 293T/17 SF] (Source: ATCC® ACS-4500™)
2) CEM-GFP cells (Source: AIDS reagents program; Catalogue # 3655)
3) MON cells are derived from an extracranial rhabdoid tumor carrying biallelic deletions of INI1 gene (INI1-/-) [a gift of Dr. Olivier Delattre, Institut Curie (Reference: Versteege I, Sevenet N, Lange J, Rousseau-Merck MF, Ambros P, Handgretinger R, Aurias A, Delattre O: Truncating mutations of hSNF5/INI1 in aggressive paediatric cancer. Nature 1998, 394(6689):203-206]

Authentication

293T cell line has been authenticated by the provider (ATCC). MON cells have been authenticated by sequencing the INI1 locus and found to be deleted (Dr. Delattre). We used all these cell lines soon after receiving them and these cell lines were used up to 10-12 passages only. These cell lines were also authenticated by their functional and morphological characterization in our laboratory. For example, CEM-GFP cells are T-cell lines harboring LTR-GFP. When these cells are infected by HIV-1, the cells turn green due to activation of GFP. MON cells are deleted of INI1 gene, and hence Western analysis of the cell lysates with anti-INI1 antibody shows the lack of INI1 protein.

Mycoplasma contamination

All cells were routinely tested for Mycoplasma contamination and they all were found to be Mycoplasma negative.

Commonly misidentified lines
(See ICLAC register)

None of the cell lines used were commonly misidentified.
